# Long-Term Treatment of Clonidine, Atenolol, Amlodipine and Dihydrochlorothiazide, but Not Enalapril, Impairs the Sexual Function in Male Spontaneously Hypertensive Rats

**DOI:** 10.1371/journal.pone.0116155

**Published:** 2015-01-23

**Authors:** Li-Li Lin, Dong Wang, Wei Wang, Yan-Qiong Cheng, Ding-Feng Su, Ai-Jun Liu

**Affiliations:** 1 Department of Pharmacology, Wuxi Higher Health Vocational Technology School, Wuxi, Jiangsu, China; 2 Department of Pharmacology, School of Pharmacy, Second Military Medical University, Shanghai, China; 3 Department of Pharmacy Administration, Chinese PLA General Hospital, Beijing, China; 4 Department of urinary surgery, Changhai Hospital, Second Military Medical University, Shanghai, China; VCU, UNITED STATES

## Abstract

This study was designed to investigate the impact of representative antihypertensive drugs of 5 classes on the sexual function in male spontaneously hypertensive rats (SHR) at doses that achieved similar blood pressure (BP) reduction. The experiment was performed in 6 groups of male SHR. The dose are 20 μg/kg/day for clonidine, 3 mg/kg/day for enalapril, 20 mg/kg/day for atenolol, 2 mg/kg/day for amlodipine, and 10 mg/kg/day for dihydrochlorothiazide. SHR were treated for 3 months, and then the penile erection and sexual behavior were detected. After BP recording, SHR were killed to evaluate the organ-damage, weight of accessory sex organs and levels of follicle-stimulating hormone (FSH), luteinizing hormone (LH) and testosterone in serum. Five drugs had the similar efficacy on BP reduction. All drugs except of enalapril, significantly prolonged the mount latency, and decreased the mount frequency (*P*<0.05). Clonidine also reduced the conception rate (45% vs. 80% in control group, *P*<0.05). Amlodipine and dihydrochlorothiazide significantly increased the testosterone level (0.79±0.30, 0.80±0.34 vs. 0.49±0.20 in control group, unit: ng/dl, *P*<0.05). Enalapril, atenolol and amlodipine also significantly decreased the BP variability (systolic, 8.2±2.5, 7.6±1.8, 8.9±2.0 vs. 12.2±3.8 in control group, unit: mm Hg). All these drugs significantly decreased the organ-damage (*P*<0.05). In conclusion, long-term treatment with 5 common antihypertensive drugs possessed obvious organ protection in SHR. Clonidine, atenolol, amlodipine and dihydrochlorothiazide, but not enalapril, impair sexual function.

## Introduction

Decreased sexual activity and function is very common in patients with cardiovascular disease [1, 2]. Hypertension is one of the most important risk factors for sexual dysfunction, and around 30% of hypertension patients complain of erectile dysfunction [3]. So blood pressure (BP) control might be useful to decrease the risk of erectile dysfunction [4–6]. Indeed, recently, a clinical study reports that BP control by β-blockers is associated with the lower prevalence of sexual dysfunction, independent of age, cardiovascular disease, and medical treatments and that beneficial effect of BP control is greater in older patients.^4^ Although some data showed that angiotensin Ⅱ antagonists might be beneficial for erectile function and sexual activity [5, 6], there are no good direct data to recommend a specific class of cardiovascular drug to improve sexual function in patients with cardiovascular disease [1]. On the contrary, many antihypertensive drugs, such as diuretics, some classical β-blockers and centrally acting sympatholytic drugs have been implicated in causing sexual dysfunction [7, 8]. Moreover, in medical practice, physicians frequently attribute sexual problems to the antihypertensive drug and modify or discontinue medication regimens. The side effect of antihypertensive hypertensive drugs might destroy the benefit of BP control on sexual function. So both BP control and the side effect of different antihypertensive drugs should be considered to improve the patients’ adherence.

In this article, we used male SHR, to compare the effects of 5 antihypertensive drugs (representing 5 classes of antihypertensive agents), at doses that achieved similar BP control.

## Methods

### Animals

SHR were provided by the animal center of Second Military Medical University. Female Sprague-Dawley (SD) rats were purchased from Sino-British SIPPR/BK Lab Animal Ltd. (Shanghai, China) to induce the penile erection of male rats and get the parameters for sexual behavior. All rats were housed in controlled temperature (23 to 25°C) and lighting (8:00 AM to 8:00 PM light, 8:00 PM to 8:00 AM dark) and with free access to standard food and drinking water. All animal experiments were approved by the Administrative Committee of Experimental Animal Care and Use of Second Military Medical University, and conformed to the National Institute of Health guidelines on the ethical use of animals.

### Drugs and drug administration

Antihypertensive drugs used in this study are as follows: Clonidine (Sigma Chemical Co, St Louis, MO, USA), enalapril (Jiangsu Hengrui Pharmaceutical Co. Ltd, Lianyungang, Jiangsu, China), atenolol (Shanghai Second Pharmaceutical Co. Ltd, Shanghai, China), amlodipine (Nanjing Pharmaceutical Co. Ltd, Nanjing, Jiangsu, China), China), and dihydrochlorothiazide (Shanghai Xinyi Pharmaceutical Co. Ltd, Shanghai, China).

To select the proper dose of different antihypertensive drugs for the similar efficacy on BR reduction, SHR were randomly divided into 11 groups (n = 5 per group) and respectively given hypertensive drugs with 2 doses: clonidine (10 and 20 μg/kg), enalapril (1.5 and 3 mg/kg), atenolol (10 and 20 mg/kg), amlodipine (1 and 2 mg/kg), and dihydrochlorothiazide (5 and 10 mg/kg) according to references [9–11]. The drugs were dissolved in 0.8% carboxymethylcellulose sodium (CMC) and given via a catheter inserted into the stomach at the same time as aortic catheter implantation. Rats in control group were only given the 0.8% CMC vehicle. After approximately 4 h habituation (from 8:00 AM to 12:00 PM), the BP signal was digitized by a microcomputer beat-to-beat. Systolic BP (SBP), diastolic BP (DBP) values from every heart beat were determined online. The mean values of these parameters during 1 h before administration (from 12:00 PM to 13:00 PM) and 6 h after administration (from 13:00 to 19:00) for each rat were calculated by the BP recording system (MPA-HBBS; Shanghai Alcott Biotech Co, Ltd, Shanghai, China) as previously described [12–14]. All the higher doses of these five drugs have the similar efficacy on BP reduction (by about 20 mm Hg reduction of SBP).

Long-term studies were performed in 6 groups of male SHR (n = 10 in each group). Clonidine, enalapril, atenolol, amlodipine and dihydrochlorothiazide were mixed in the rat chow, respectively. The content of drugs in the rat chow was calculated according to the chow consumption. The concentration of the drugs in the rat chow were adjusted to 20 μg/kg/day for clonidine, 3 mg/kg/day for enalapril, 20 mg/kg/day for atenolol, 2 mg/kg/day for amlodipine, 10 mg/kg/d for dihydrochlorothiazide according to the results of the former acute experiment. The control SHR group received the same diet without any drugs.

### Penile erection

Penile erection was determined using the method reported [15, 16]. The rats in each group were placed in transparent cages and continuously observed for a period of 30 min. The cage was divided in half by 2 sheets of plastic mesh, preventing contact but allowing auditory, visual, and olfactory stimulation. After 5 min adaptation period, the test was started by placing an estrus female SD rat on the other side of the cage. Cages were cleaned before shifting the animals of different groups. The number of erection was recorded. Erection in rats was marked by the visibility of the penis out of its sheath or by grooming of the penis. The observation was recorded before and after 3-month treatment. Penile erection index was determined by multiplying the percentage of rats exhibiting at least one episode of penile erection during 30 min observation period with the mean number of penile erection. Animals without penile erection before drugs treatment were discarded.

### Parameters for sexual behavior analysis

After 3-month drug administration, every male SHR was put in a single cage with 2 female SD rats (during non-estrus) aged 3 months. The mounting behavior were observed for 30 min (from 6:00 PM to 6:30 PM) by using digital counters with infrared sensors (Shanghai Jiliang software technology Co., Ltd, Shanghai, China) as references reported [15–17].

Mounting behavior: Mount frequency was determined by counting the number of mounts in given period of observation. Mount latency was calculated as the time lapse from the introduction of female to the occurrence of first mount. After one week copulation, the female rats were separated, and the conception rate was recorded.

Upon the completion of these experiment, BP was recorded for 6 h (13:00 to 19:00), and then SBP, DBP and heart rate of rats were continuously recorded in conscious freely moving rats. The average value was used as an index of BP, and the standard deviation of beat-to-beat BP values as an index of BP variability (BPV) including systolic BPV (SBPV) and diastolic BPV (DBPV). The same method was used for the calculation of heart rate variability (HRV) [18, 19]. Noninvasive BP measurements were made using a tail-cuff system (Alcott Biotech) as described method [18]. After 3 days of training, each rat was assessed for a minimum of 3 times per session.

### Morphological examination

Morphological examinations were performed after BP recording. The animal was weighed and anesthetized. The blood samples were collected for the detection of serum hormone levels. Then the thoracic and peritoneal cavities were immediately opened. The heart was excised and rinsed in cold physiological saline. The ventricle (including left and right ventricle) was isolated and weighed. At the same time, the aorta was cleaned of adhering fat and connective tissue. Just below the branch of the left subclavicular artery, a 30-mm-long segment of thoracic aorta was harvested and weighed. The accessory sex organs such as testis, epididymis, seminal vesicle and prostate were also dissected quickly. Ratios of ventricular weight to body weight (VW/BW), left ventricular weight to body weight (LVW/BW) and aortic weight to the length of aorta (AW/length) were calculated. The ratios of accessory sex organ weight to body weight, including testis weight to body weight (TW/BW), epididymides weight to body weight (EW/BW), seminal vesicles weight to body weight (SVW/BW) and prostate weight to body weight (PW/BW) were also calculated as the percentage of accessory sex.

### The detection of follicle-stimulating hormone (FSH), luteinizing hormone (LH) and testosterone

Serum was collected, and the FHS, LH and testosterone levels were detected with radio-immunity kits (ShiRuiKe Biotechnology (Shanghai) Co., Ltd, Shanghai, China).

### Statistical analysis

Investigators were blind to the procedures during BP recording, sexual activity measurement and morphological examination. Data are expressed as mean ± standard deviation (SD). Data are analyzed with 2-tailed Student’s t test (between before and after drugs administration) and one-way analysis of variance (ANOVA) followed by dunnett *t*-testing or LSD *post-hoc* testing. Chi-square test is used to estimate the conception rate. *P*<0.05 was considered statistically significant.

## Results

### Effects of long term treatment of 5 antihypertensive drugs on body weight and BP in SHR

**Figure 1:**
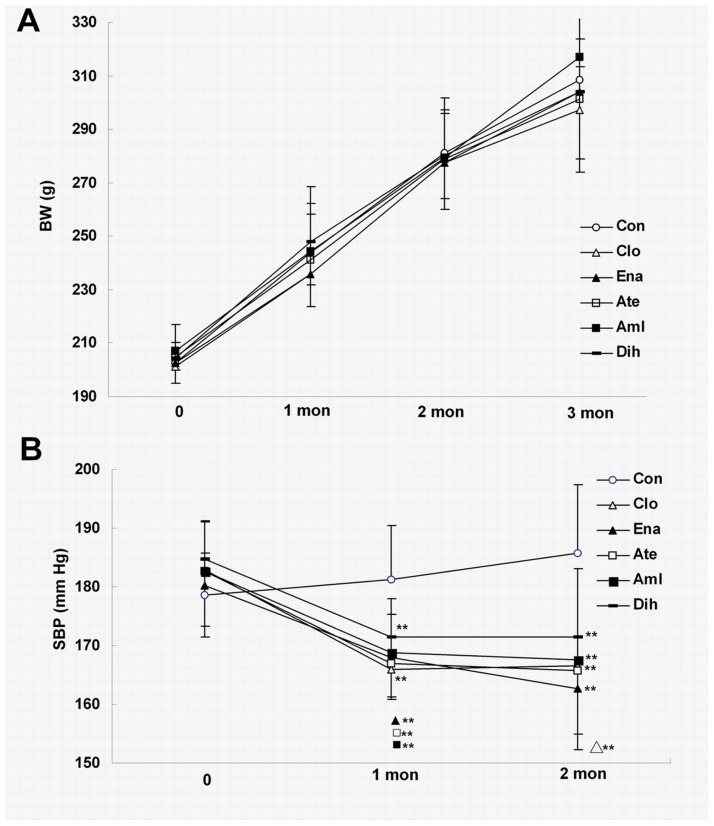
Effect of 3-month treatment of 5 antihypertensive drugs on the body weight and SBP level of SHR. The changes of body weight (A) and SBP level (B) during 3-month treatment of 5 antihypertensive drugs. Data are shown as mean ± SD and analyzed by ANOVA followed by dunnett *t*-testing. **P<0.01 vs. control. Abbreviation: BW, body weight; Con, control; Clo, clonidine; Ena, enalapril; Ate, atenolol; Aml, amlodipine; Dih, dihydrochlorothiazide.

In a period of 3 months after antihypertensive drugs manipulation starting from 3 months of age, the body weight increased by about 100 g in the 6 different groups (Fig. 1A). There is no difference in the body weight of 6 different groups. Noninvasive BP measurements: Compared to control group, treatment by antihypertensive drugs for 1 month significantly decreased the SBP level (Clonidine, 167±11 mm Hg; enalapril, 168±7 mm Hg, atenolol: 167±6 mm Hg; amlodipine, 168±6 mm Hg and dihydrochlorothiazide, 171±12 mm Hg, vs. 181±9 mm Hg in control, *P*<0.01). Similar results were obtained with 2-month treatment (Fig. 1B). There’s no difference in SBP level in 5 drugs treatment groups.

### Effects on the penile erection and sexual behavior in SHR

**Figure 2:**
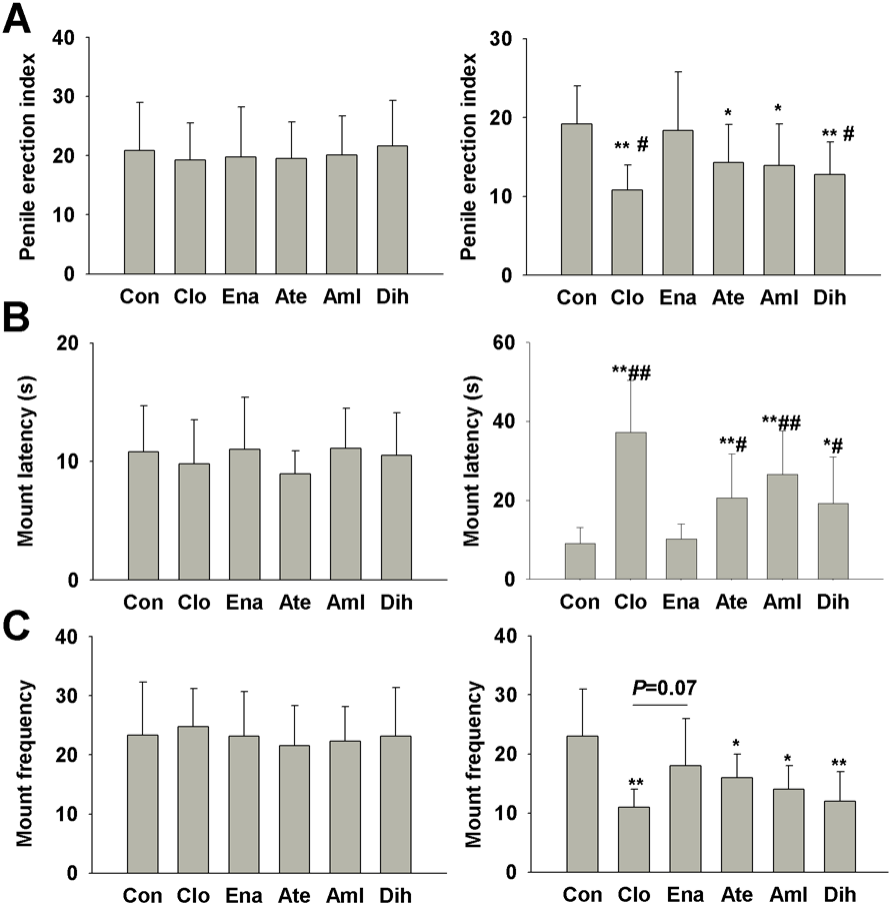
Effects of 3-month treatment of 5 different antihypertensive drugs on the penile erection and sexual behavior of male SHR. (A) Penile erection was determined for 30 min. The rat was placed in a transparent cage which was divided in half by 2 sheets. An estrus female SD rat was placed on the other side of the cage. The number of erections were recorded before (left) and after 3-month drug treatment (right). (B) and (C) Every male SHR was put in a single cage with 2 female SD rats (during non-estrus). The mount latency (B) and mount frequency (C) were recorded for 30 min before (left) and after 3-month drug treatment (right). Data are presented as mean ± SD and analyzed by ANOVA followed by LSD *post-hoc* testing. *P<0.05, **P<0.01 vs. control. # *P*<0.05, ## *P*<0.01 vs. Ena. SHR, n = 10, non-estrus female SD rats, n = 2. Estrus female SD rats, n = 1. Abbreviation: Clo, clonidine; Ena, enalapril; Ate, atenolol; Aml, amlodipine; Dih, dihydrochlorothiazide.

Before drugs treatment, the parameters of penile erection, mount latency and frequency were recorded. There’s no difference in these 3 parameters between 6 groups (Fig. 2A, B and C, left). We also recorded the penile erection and mount behavior after 3-month drugs manipulation. Compared to the control group, the penile erection index and mount frequency were significantly decreased in male SHR treated with clonidine, atenolol, amlodipine and dihydrochlorothiazide (*P*<0.05, Fig. 2A, C right). All these 4 drugs also significantly prolonged the mount latency (*P*<0.01, Fig. 2B right). There was no significant change in penile erection index, mount latency and frequency in enalapril treatment group. Compared to enalapril, clonidine and dihydrochlorothiazide significantly decreased the penile erection index (*P*<0.05, Fig. 2A right), and clonidine, atenolol, amlodipine and dihydrochlorothiazide significantly increased the mount latency (*P*<0.05, Fig. 2B right).

### Effects of long term treatment of 5 antihypertensive drugs on BP

**Figure 3:**
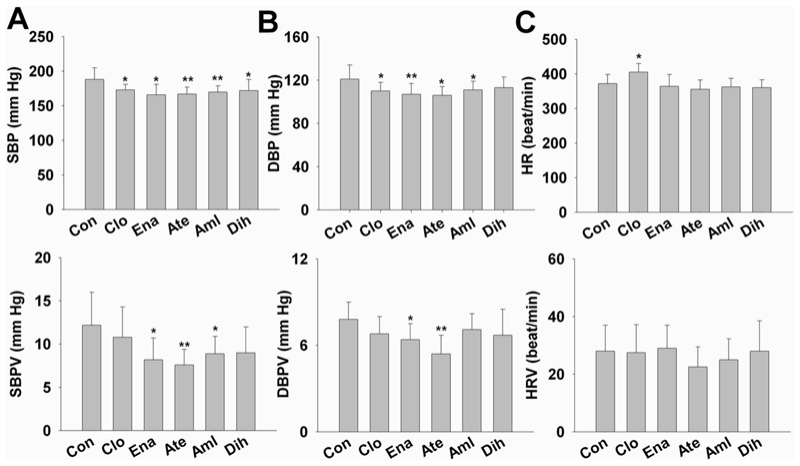
Effects of 3-month treatment of 5 different antihypertensive drugs on the hemodynamics in SHR. (A) SBP and SBPV. (B) DBP and DBPV. (C) HR and HRV. Data are presented as mean ± SD and analysized by ANOVA followed by dunnett *t*-testing. *P<0.05, **P<0.01 vs. control. Abbreviation: Con, control; Clo, clonidine; Ena, enalapril; Ate, atenolol; Aml, amlodipine; Dih, dihydrochlorothiazide; SBP, systolic blood pressure; DBP, diastolic blood pressure; SBPV, systolic blood pressure variability; DBPV, diastolic blood pressure variability; HR, heart rate; HRV, heart rate variability.

We also recorded the BP by the invasive BP recording system after 3-month drugs manipulation. Compared to control group, 3-month treatment with clonidine significantly decreased both SBP (173±8 mm Hg vs. 188±17 mm Hg in control group, *P*<0.05, Fig. 3A) and DBP (110±8 mm Hg vs. 121±13 mm Hg, *P*<0.05, Fig. 3B), and increased the heart rate (405±25 vs. 372 ±26, *P*<0.05, Fig. 3C). Dihydrochlorothiazide only significantly decreased the SBP level (*P*<0.05, Fig. 3A), and did not affect the DBP and the heart rate. Enalapril, atenolol and amlodipine significantly decreased levels of SBP, DBP, and also significantly decreased the BPV (Fig. 3A and 3B). Dihydrochlorothiazide and clonidine did not affect the SBPV, DBPV. All these drugs except of clonidine, did not affected the heart rate and HRV (Fig. 3C).

### Effects on organ damage in SHR

**Figure 4:**
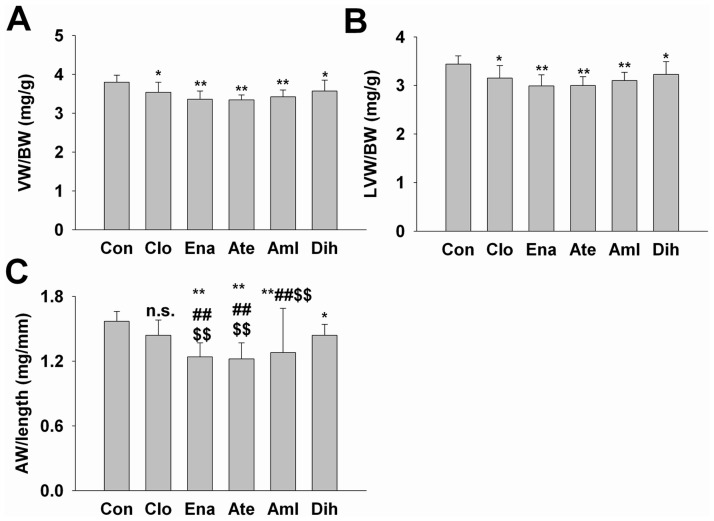
Effects of 3-month treatment of 5 different antihypertensive drugs on the end-organ damage of ventricle and aorta in SHR. (A), (B) and (C), Data are shown as mean ± SD. Data are analyzed by ANOVA followed by LSD *post-hoc* testing. *P < 0.05, **P < 0.01 vs. Con; ##*P* < 0.01 vs. Clo; $$*P* < 0.01 vs. Dih; n.s., not significant, n = 10. Abbreviation: VW, ventricular weight; BW, body weight; LVW, Left ventricular weight; AW, aortic weight; Clo, clonidine; Ena, enalapril; Ate, atenolol; Aml, amlodipine; Dih, dihydrochlorothiazide.

Compared to control group, 3-month treatment of 5 different drugs significantly decreased the VW/BW and LVW/BW (*P*<0.05, Fig. 4A and B). Enalapril, atenolol, amlodipine and dihydrochlorothiazide also significantly decreased AW/length, while clonidine did not change AW/length (*P*>0.05, Fig. 4C). Compared with clonidine or dihydrochlorothiazide, enalapril, atenolol and amlodipine also significantly decreased AW/length (*P*<0.01, Fig. 4C). These data indicated that all 5 drugs can decrease the cardic hypertrophy.

### Effects on weight of accessory sex organs and the hormone level in male SHR and conception rate in female

**Figure 5:**
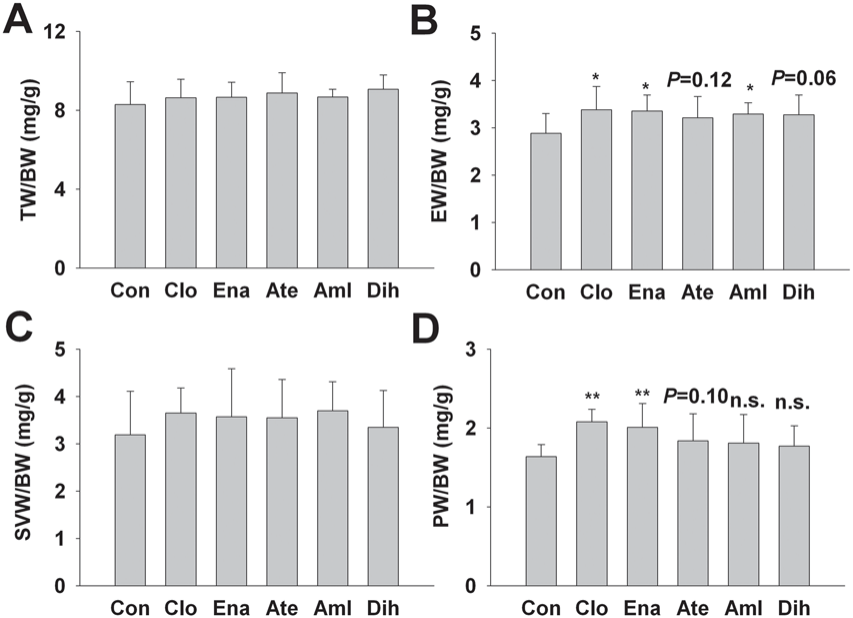
Effects of 3-month treatment of 5 different antihypertensive drugs on the weight of accessory sex organ in SHR. Data are shown as mean ± SD. Data are analyzed by ANOVA followed by dunnett *t*-testing. *P < 0.05, **P < 0.01 vs. Con; n.s., not significant, n = 10. Abbreviation: TW, testis weight; BW, body weight; EW, epididymides weight; SVW, seminal vesicles weight; PW, prostate weight; Con, control; Clo, clonidine; Ena, enalapril; Ate, atenolol; Aml, amlodipine; Dih, dihydrochlorothiazide.

Compared to control group, drugs treatment did not affect the testis index (TW/BW) and seminal vesicles (SVW/BW) of 6 groups (*P*>0.05, Fig. 5A, 5C). Compared to control group, clonidine, enalapril and amlodipine significantly increased epididymides index (EW/BW) (*P*<0.05, Fig. 5B). For prostate index, clonidine and enalapril significantly increased PW/BW (*P*<0.01, Fig. 5D).

**Figure 6:**
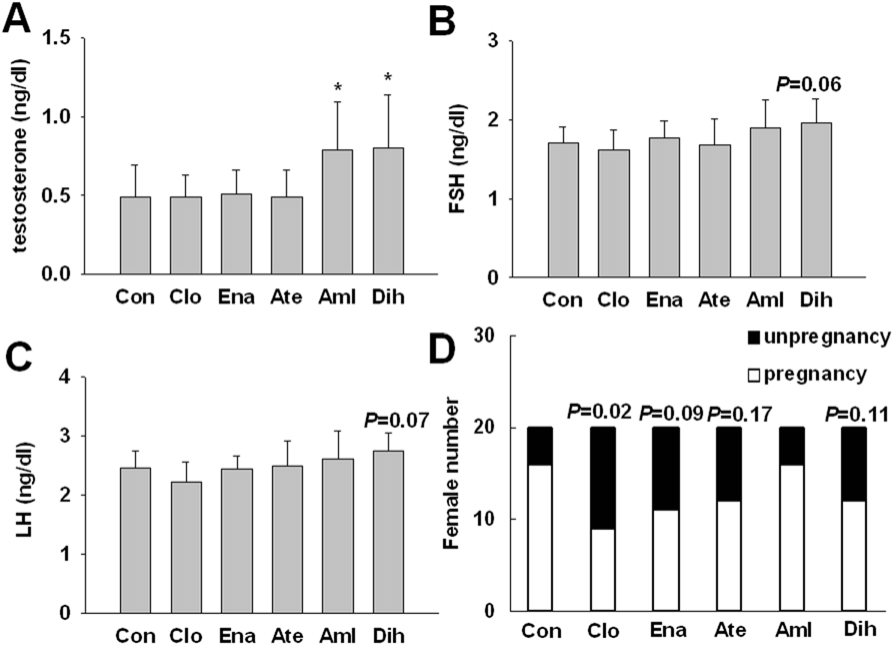
Effects of 3-month treatment of 5 different antihypertensive drugs on the sexual hormone levels and the conception rate of SHR. (A), (B) and (C), Data are shown as mean ± SD. Data are analyzed by ANOVA followed by dunnett *t*-testing. *P < 0.05, vs. Con. (D), every male SHR was put in a single cage with 2 female SD rats. After one week copulation, the conception rate of the female rats was recorded (D). Chi-square test is used to estimate the conception rate. SHR, n = 10, Female SD rats, n = 20. Abbreviation: Con, control; Clo, clonidine; Ena, enalapril; Ate, atenolol; Aml, amlodipine; Dih, dihydrochlorothiazide; FSH, follicle-stimulating hormone; LH. luteinizing hormone.

Compared to control group, only amlodipine and dihydrochlorothiazide significantly increased the testosterone level (*P*<0.05, Fig. 6A) All 5 drugs did not affect the levels of FSH and LH (*P*>0.05, Fig. 6B and 6C).

Compared to control group, the conception rate was significantly decreased by clonidine (45% vs. 80% in control group, *P*<0.05, Fig. 6D). Enalapril, atenolol and dihydrochlorothiazide amlodipine, also slightly affected the conception rate but not significantly (*P*>0.05). Amlodipine did not affect the conception rate.

## Discussion

Although recent studies and reviews have not found clear relationships between contemporary antihypertensive drugs and sexual activity [20–23]; many reports also show that numerous classes of cardiovascular drugs have been implicated in causing erectile dysfunction [7, 8, 24]. The sexual activity of treated hypertensive patients was less prevalent and the sexual problems were more prevalent [25]. This discrepancy might be partly related to double function of the antihypertensive: its detrimental or beneficial effect on the sexual activity. As BP control was associated with a lower prevalence of sexual dysfunction independently [4], the balance of BP control and the side effect of antihypertensive drugs should both be considered to evaluate their effects on sexual activity.

In this study, we chose five commonly clinical antihypertensive drugs with similar BP reduction, to compare their effects on sexual function. The BP control was about 15 mm Hg by adjusting the dose to exclude the factor of BP. Our data showed that only enalapril did not affect the sexual activity. We also found that clonidine significantly decreased the conception rate.

Beta-blockers, calcium channel blocker and angiotension converting enzyme (ACE) inhibitors are recommended by WHO because of their definitely antihypertensive efficacy, few adverse side effects, organ protection and risk factors reduction related to hypertension [9, 26, 27]. Among these beneficial effects of antihypertensive drugs, BPV reduction and organ protection are very important factors. Data even show that BPV may be more important than the BP level in the determination of cardiac damage, renal lesions and aortic hypertrophy [28]. Because sexual dysfunction is associated with cardiovascular risk factors and cardiovascular disease [29], the sexual function may be improved by the organ protection. In this study, we found the protective effect of enalapril, atenolol and amlodipine against the cardiac and aortic hypertrophy. The vascular protective effect of these 3 drugs is better than clonidine and dihydrochlorothiazide. This might partly explain the different manifestations in sexual function between clonidine, dihydrochlorothiazide and other 3 drugs.

Clonidine is a kind of central antihypertensive drug. Because of symptomatic side effects, the use of clonidine has been limited. The sexual problems of clonidine, especially in men, are also prominent [30]. But in some area of China, its compound preparation is also been used because of its cheap price and antihypertensive efficacy. In our present data, we found that clonidine affected the sexual function more seriously. It even decreased the conception rate. So this side effect of traditional old drug should be carefully considered, especially for young patients with hypertension.

The other old antihypertensive drug is thiazide diuretics. Data show that sexual dysfunction is frequently encountered when thiazide diuretics are used in combination with other drugs [31, 32]. Indeed, in this study, although dihydrochlorothiazide significantly increased the testosterone level and decreased the organ damage, the administration of dihydrochlorothiazide alone also significantly decreased the sexual activity.

Besides clonidine and dihydrochlorothiazide, we also found the detrimental effect of atenolol on sexual activity. But recently the negative effect of β-blockers on sexual activity has been recently debated [22, 31]. On the one hand, an analysis of 6 studies involving almost 15 000 people found β-blocker therapy increased the annual reported rate of sexual dysfunction by only 5 reports per 1000 patients and the annual reported rate of impotence by only 3 per 1000 patients [33]. Some reports even suggest that β-blockers induced erectile dysfunction seems to be perceived and not real [31]. On the other hand, some carefully designed, randomized crossover studies provided strong evidence for a detrimental role of β-blockers on sexual function [31]. Both traditional and new β-blockers share the detrimental effect on sexual function [31]. According to our data and the clinical studies, the negative effect of β-blockers on sexual should not be negated.

The nerves and endothelium of sinusoids and vessels in the penis produce and release transmitters. These transmitters control the contractile state of the penile smooth muscles. Nitric oxide (NO) might be the most important transmitter for erection [34, 35].Erectile function is mainly dependent on NO production by penile endothelium. Loss of the functional integrity of the endothelium and subsequent endothelial dysfunction plays a pivotal role in the occurrence of sexual dysfunction [34]. So endothelial dysfunction is an important feature of sexual dysfunction associated with reduced plasma NO levels. The detection of NO level might be an important method to evaluate the influence of antihypertensive drugs on the erectile function.

Contrary to NO, Ang Ⅱ is an important factor to mediate contraction. Ang Ⅱcontributes to maintenance of the penis in a flaccid state [34]. It could be expected that drugs reducing the formation or action of Ang Ⅱ, such as ACE inhibitors or angiotensin receptor blockers (ARBs), should improve erectile responses. In SHR, enalapril induced structural remodeling of the penile vasculature and ameliorated blood inflow to the corpora cavernosa [36]. Captopril improved erectile function of hypertensive and normotensive aged rats [37].

A few clinical studies have suggested that treatment with ARBs or ACE inhibitors may improve erectile function [34]. However, a recent substudy of ONTARGET/TRANSCEND trials concluded that treatment with telmisaran, ramipril and their combination did not ameliorate erectile dysfunction. These drugs did not prevent new-onset erectile dysfunction [29]. We got the similar information about enalapril. Enalapril did not improve the sexual function. But more importantly, among these 5 drugs, it’s the only drug that did not ameliorate the sexual function. It is obvious that the role of the renin-angiotensin system in the corpora cavernosa is more complicated that was previously believed.

Although recent result suggested that the felodipine combined with ARB improved sexual function in hypertensive women [38], more available evidences suggested calcium channel blockers (CCB) may be neutral with respect to the endpoint of sexual function [8]. Our data even showed the negative effect of amlodipine on sexual activity. This result needs more large clinical trials to evaluate specifically the role of CCB on sexual function.

Besides Ang Ⅱ, increased production of inflammatory cytokines is another important factor to mediate contraction. It’s also an important biochemical marker of endothelial dysfunction. A candidate factor is tumor necrosis factor (TNF-α) [34]. TNF-α can play an important role in cardiovascular disease, mainly because of its direct effects on the vasculature, and may also be involved in sexual dysfunction. TNF-α impaired endothelium-dependent and NO mediated vasodilation in various vascular beds. TNF-α may also show a key function in mediating endothelial dysfunction in erectile dysfunction [34].

Blockade of TNF-α action may theoretically represent an alternative therapeutic approach for erectile dysfunction, especially in pathological conditions associated with increased levels of this cytokine [34]. Recently, Ciccone et al. evaluated the role of a new biochemical pathway (soluble suppression of tumorigenicity 2 (sST2) actions) as able to influence vascular function and cardiovascular risk. It can reduce the production and activation of NF-kB, thus it would reduce inflammatory response [39]. Whether TNF-α can be a treatment alternative or protection of sST2 activation in such cases of sexual dysfunction remains to be established.

In conclusion, with the similar BP reduction, long term treatment with 5 common antihypertensive drugs possessed obvious organ protection in hypertension rats. Clonidine, atenolol, amlodipine and dihydrochlorothiazide but not enalapril impaired sexual function.
